# Case Report: Wellens syndrome in acute total occlusion saved by collateral

**DOI:** 10.12688/f1000research.125820.1

**Published:** 2022-12-12

**Authors:** Mochamad Yusuf Alsagaff, Tony Santoso Putra, Bagus Putra Dharma Khrisna, Ricardo Adrian Nugraha

**Affiliations:** 1 Department of Cardiology and Vascular Medicine, Faculty of Medicine, Universitas Airlangga, Dr. Soetomo Academic General Hospital, Surabaya, East Java, 60286, Indonesia

**Keywords:** acute myocardial infarction, acute coronary syndrome, percutaneous coronary intervention, Wellens syndrome

## Abstract

**Background:** It is important and challenging to distinguish between acute myocardial infarction and Wellens syndrome due to its time to intervention. Difficulties in differentiating between subtypes could mean the patients are overtreated or receive undertreatment.

**Case report:** A 57-year-old man was referred to our emergency ward with acute onset of chest pain. Electrocardiographic (ECG) changes were suggestive of Wellens syndrome type A. Nitroglycerin was administrated, the patient's chest pain disappeared, and we planned an early invasive strategy. He had a previous documented ECG before he went for catheterization and based on the second ECG changes were suggestive of an ST elevation. As the result of the invasive strategy, it was found that there was single-vessel disease, near total occlusion in the middle of the left anterior descending artery (LAD) with collateral from the right coronary artery. After two days of observation in the Intensive Cardiovascular Care Unit (ICCU), the patient improved and was transferred to the Low Care Unit.

**Conclusions:** The case highlights Wellens syndrome in acute total occlusion with collateral artery.

## Introduction

Wellens syndrome is characterized by specific ECG profiles in the precordial lead, especially in the T-wave segment, which is associated with critical stenosis of the proximal left anterior descending (LAD) coronary artery. ^1^ The incidence rate is 10–15% of all patients with acute coronary syndrome. ^2^ Moreover, the presence of coronary collateral circulation can change an ECG picture that should be an ST-elevated myocardial infarction due to total or near total occlusion of the left anterior descending artery, turning into an ECG picture of Wellens syndrome.

In this case, we present Wellens syndrome in acute total occlusion saved by collateral. Significant collateral circulation is believed to improve clinical outcomes, especially in patients with acute coronary syndrome.

## Case report

A 57-year-old man presented at Dr. Soetomo General Hospital, Surabaya, Indonesia, with sudden chest pain. He had a history of uncontrolled type 2 diabetes mellitus, uncontrolled hypertension, hypercholesterolemia, and a recent history of angina three years prior without undergoing a revascularization procedure. There was no history of cocaine use. He was brought to the emergency department with acute onset of substernal chest pain and diaphoresis. The chest pain started two hours before he was brought to the emergency ward. His chest pain score was four on the visual analogue scale (VAS). Nitroglycerin was administrated then the patient's chest pain disappeared. On physical examination on the emergency ward, he was afebrile with a heart rate of 99, blood pressure of 170/100 mmHg, respiratory rate of 22 breaths per minute, and oxygen saturation of 95% in room air. He was overweight with a pained grunt and he occasionally clutched his chest. Physical examination was unremarkable.

### Timeline

**Figure 1.**
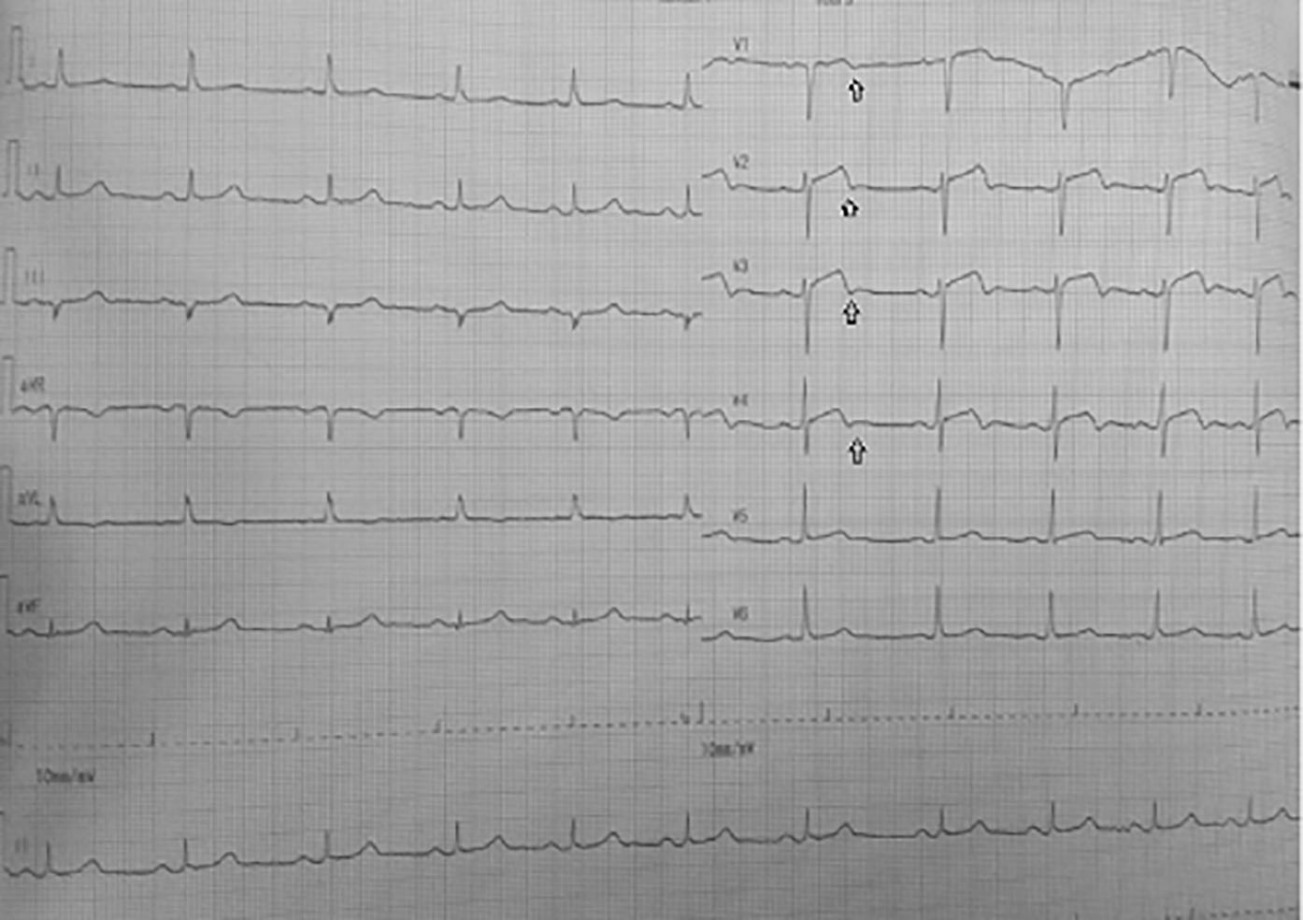
ECG: Biphasic T wave in V1–V4 (Wellens syndrome type A) and slight ST elevation in lead V2–V4.

**Figure 2.**
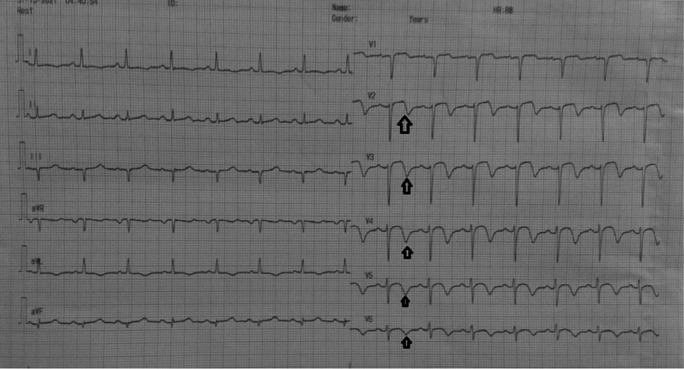
ECG: T-wave inversion in leads V2–V5 and slight ST-segment elevation in lead V2–V3.

On day one, the patient was admitted to our emergency ward due to abrupt and sudden onset chest pain with VAS 4/10, that was not relieved by nitroglycerin. ECG showed a biphasic T wave in V2–V4 (Wellens A) ( Figure 1). The patient was diagnosed with non-ST elevation myocardial infarction. On day two, transthoracic echocardiography revealed regional wall motion abnormality with reduced ejection fraction. ECG changed into an anterior acute ST-elevation myocardial infarction. Then, we planned to perform early invasive strategy. Coronary angiography revealed a total occlusion in the middle left anterior descending coronary artery. We performed percutaneous coronary intervention and stenting in the lesion. On day four, the patient improved significantly during the critical period and was transferred to low care. On day five, ECG revealed an inverted T wave in V2–V6 (Wellens B) ( Figure 2), and then the patient was discharged without any sequelae.

### Investigations

Significant laboratory findings suggested elevated troponin I levels 2,058 ng/ml (<0.02), HbA1c 8.5% (<6.5%), and complete blood count, renal function test, liver function test and serum electrolyte were within normal limits. ECG in the emergency department ( Figure 1) showed an ST segment of less than 1 mm, and there was a biphasic T wave in V2–V4 (Wellens type A) ( Figure 2); echocardiography was therefore performed showed ejection fraction was 48%, there was LV dilatation (LVIDd 6.1cm) and eccentric left ventricular hypertrophy (LVdMI: 132.62 g/m2; RWT: 0.320); from segmental analysis, it was found that there was hypokinetic in the anteroseptal (B–M) region, septal (A) region; others regions were within normal limits.

**Figure 3.**
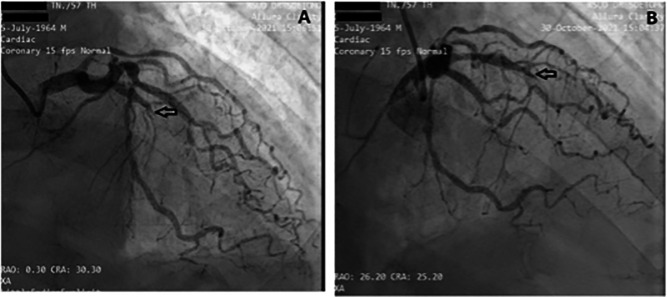
Angiography: A and B LAD projection view showed total occlusion of 100% in the middle LAD. The left circumflex artery (LCx) had non-significant stenosis of 40% proximal and 65% distal LCx.

**Figure 4.**
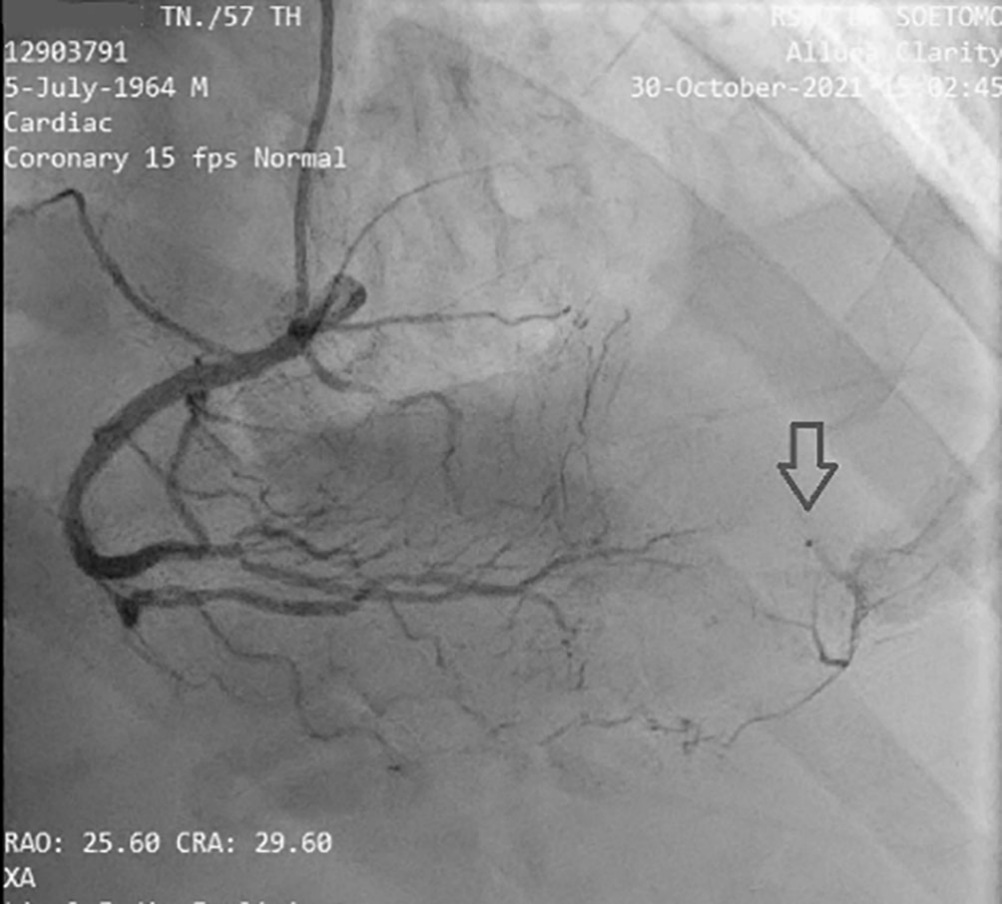
Angiography RCA projection View RAO, CRA. It is revealed that there is grade II collateral from distal RCA to distal LAD. (Notes: RAO CRA = right anterior oblique + cranial view, a radiographic projection).

Before the invasive strategy was done, the patient had another ECG eight hours after the first ECG, based on the second ( Figure 2). The ECG showed ST-segment elevation changes that were greater than 1 mm. The early invasive strategy ( Figure 3 and Figure 4; Extended data: Video 1 and Video 2) ^3^^,^^4^ showed that the left anterior descending artery (LAD) had total occlusion of 100% in the middle LAD ( Figure 3). The left circumflex artery (LCx) had non-significant stenosis of 40% proximal and 65% distal, and right coronary artery (RCA) had non-significant stenosis of 55% distal. The RCA had grade 2 collateral arteries that supply blood to the mid LAD ( Figure 4).

### Differential diagnosis

Right Bundle Branch Block (RBBB) **:** the ECG shows an inverted T wave, RsR’ in lead V1–V3, QRS duration is >130 ms.

Pulmonary embolism has symptoms of chest pain, but the ECG shows S1Q3T3. It means the presence of an S wave in lead I (indicating a rightward shift of the QRS axis) with a Q wave and T inversion in lead III.

Cocaine users have ECG patterns like Wellens, called “pseudo-Wellens syndrome”, due to vasospasm of the coronary arteries.

### Treatment

In accordance with the 2020 European Society of Cardiology Guidelines for Acute Coronary Syndrome, ^5^ our patient received an acetylsalicylic acid loading dose of 300 mg followed by 100 mg once daily, high dose statin with Atorvastatin 40 mg once daily at night, Bisoprolol 2.5 mg once daily, Ramipril 5 mg once daily in the morning, Fondaparinux injection 2.5 mg subcutaneous once daily, IV nitroglycerin 20 mcg/minute for hypertension; the patient’s angina subsided. He was also receiving basal insulin bolus for his diabetes. An early invasive strategy was carried out and the patient was then moved to the ICCU.

### Outcome and follow up

Our patient improved significantly during the critical period. After two days in the ICCU, he had no complaints. Based on physical examination, he was afebrile. His heart rate was 70 beats per minute, with blood pressure 123/70 mmHg, respiratory rate 18 breaths per minute, and peripheral oxygen saturation was 95% with a nasal cannula of three litres per minute. Our patient was no longer using nitroglycerin pumps and anticoagulants, and he was transferred to the low-care unit. After three days in the hospital, the results of the ECG examination showed an inverted T wave in V2–V6, and then the patient was discharged without any sequelae. After one month, the patient was controlled for the disease without any sequelae.

## Discussion

Wellens ECG pattern is commonly found in a patient with total or near total occlusion in the proximal left anterior descending coronary artery. It is commonly associated with NSTE-ACS, followed by new onset angina CCS III-IV or crescendo angina, without increased cardiac markers. ^6^ The spontaneous transformation from Wellens type A ECG pattern into Wellens type B ECG pattern is a rare case. It reflects a pattern of electrocardiography (ECG) that sometimes changes due to occlusion and spontaneous reperfusion from the collateral coronary artery. Wellens type A and type B are particular for critical, proximal stenosis of the left anterior descending (LAD) coronary artery. ^7^ Criteria for diagnosing Wellens syndrome include all of the following ^8^^,^^9^:

• Wellens type A describes a pattern of ECG that shows biphasic T wave in the lead V2–V4
• Wellens type B describes a pattern of ECG that shows T inverted in the lead V2–V4
• History of angina
• ECG without Q wave
• Normal or minimally elevated troponin levels
• ST segment isoelectric or minimally elevated (<1 mm)

In this case, the first ECG showed a biphasic T wave in lead V1–V4 (Wellens type A) and it was minimally elevated (<1 mm). After a few hours before the invasive procedure, the second ECG showed a change in ST-segment elevation of more than 1 mm. In this case, the patient's ECG and troponin level showed Wellens syndrome. However, after a few hours the patient's ECG showed changes, namely an increase in elevation in the ST segment (>1 mm). An abnormal T wave ischemic pattern may persist, remaining between hours to weeks, even when the patient is asymptomatic without chest pain. ^7^ T wave abnormalities can be normalized or evolved into ST-segment elevation in the symptomatic patient with Wellens syndrome. The mechanism responsible for these ECG findings is repolarization heterogeneity resulting from reperfusion of a briefly occluded LAD. This could explain the evolution of T-wave modifications. ^10^^,^^11^ In this case, there was complete occlusion in the middle LAD, but the patient received collateral artery supply from the RCA so that the ECG feature mimicked the critical occlusion picture in the proximal LAD.

The collateral of the coronary artery is classified into five grades. Collateral grade 0 reveals no flow between the collateral of the coronary artery. This condition can occur when several collateral arteries are visible yet not angiographically apparent at any other time. Collateral grade 1 flow reveals a barely apparent collateral coronary artery. Sometimes, there might be an unclear connection with the significant epicardial coronary artery. Collateral grade 2 flow reveals a moderately opaque collateral coronary artery but it was only present through 75% of the cardiac cycle. Collateral grade 3 flow reflects a well-opacified collateral coronary artery while the column of dye is well defined (i.e., >0.5 mm in diameter), but it was <0.7 mm wide throughout most of its length. Collateral grade 4 flow mimics collateral grade 3. The collateral is very well opacified, fills antegrade, and is very large. It was >0.7 mm in diameter throughout its length. ^12^^,^^13^

The cardiac catheterization results found that a collateral artery originating from the right coronary artery (RCA) supplied the mid-distal LAD. Therefore the heart muscle that should have been severely damaged due to not getting blood supply from the LAD could still survive due to the presence of the collateral artery. Previous data has established that enough collaterals can prevent ischemia and directly induce spontaneous reperfusion in one-third of NSTE-ACS patients. Spontaneous reperfusion with well-developed coronary collateral circulation is associated with a better prognosis in cardiovascular mortality reduction. ^14^

In ischemic conditions, collateral circulations are a good predictor of prognosis. Collateral circulation may support cardiac function and decrease cardiac mortality and morbidity rate. Collateral circulations are inter arterial anastomoses that exist neonatally and grow more significant because of many mechanisms like cytokines and growth factors induced by shear stress and ischemia and pressure gradient changes. ^15^ The collateral circulation artery may appear several weeks after occlusion. ^14^

The patient felt that his treatment at the hospital was excellent and fast, especially when he felt chest pain. After the medication was taken, his chest pain gradually disappeared. The patient was also very grateful for the direct action of inserting a catheter into the heart in less than 24 hours. The patient hopes that the publication of cases of diseases like his will help paramedics handle them in the future.

## Conclusions

This case shows Wellens syndrome, which can change into ST-elevation myocardial infarction. Wellens can also occur in the left anterior descending artery that is in total occlusion but gets its blood supply from a branch of the right coronary collateral artery. Based on this case report, an early invasive strategy is recommended, with favorable clinical outcomes.

## Consent

Written informed consent for publication of their clinical details and clinical images was obtained from the patient.

## Data Availability

All data underlying the results are available as part of the article and no additional source data are required. Figshare: Extended data for ‘Case Report: Wellens syndrome in acute total occlusion saved by collateral’. This project contains the following extended data: Video 1: Angiography revealed total occlusion at mid left anterior descending coronary artery Video 2: Angiography revealed collateral from distal right coronary artery to distal left anterior descending coronary artery Data are available under the terms of the Creative Commons Attribution 4.0 International license (CC-BY 4.0).
