# Case Report: Wellens syndrome in acute critical coronary occlusion saved by collateral

**DOI:** 10.12688/f1000research.125820.3

**Published:** 2024-07-05

**Authors:** Mochamad Yusuf Alsagaff, Tony Santoso Putra, Bagus Putra Dharma Khrisna, Ricardo Adrian Nugraha

**Affiliations:** 1 Department of Cardiology and Vascular Medicine, Faculty of Medicine, Universitas Airlangga, Dr. Soetomo Academic General Hospital, Surabaya, East Java, 60286, Indonesia

**Keywords:** acute myocardial infarction, acute coronary syndrome, percutaneous coronary intervention, Wellens syndrome

## Abstract

**Background:**

It is important and challenging to distinguish between acute myocardial infarction and Wellens syndrome due to its time to intervention. Difficulties in differentiating between subtypes could mean the patients are overtreated or receive undertreatment.

**Case report:**

A 57-year-old man was referred to our emergency ward with acute onset of chest pain. Electrocardiograms changes were suggestive of type A Wellens syndrome. Nitroglycerin was administrated, the patient's chest pain disappeared, and we planned an early invasive strategy. He had a previous documented electrocardiogram before he went for catheterization and based on the second electrocardiogram changes were suggestive of an ST-elevation. As the result of the invasive strategy, it was found that there was single-vessel disease, critical occlusion in the middle of the left anterior descending artery coronary artery with collateral from the right coronary artery. After two days of observation in the Intensive Cardiovascular Care Unit, the patient improved and was transferred to Low Care Unit.

**Conclusions:**

The case highlights Wellens syndrome in acute critical occlusion with collateral artery.

## Introduction

Acute ST-elevation myocardial infarction (STEMI) equivalents represent coronary occlusion without meeting the traditional ST-elevation criteria. It's important to recognize these patterns in a timely fashion, since STEMI equivalent patterns often require emergent or urgent percutaneous coronary intervention (PCI) to minimize morbidity and mortality. One of the most common STEMI equivalent is Wellens syndrome.

Wellens syndrome is characterized by specific an electrocardiogram profiles in the precordial lead, particularly deeply inverted or biphasic T waves in leads V2-V3, that is highly specific for critical, proximal stenosis of the left anterior descending (LAD) coronary artery. ^1^ The incidence rate is 10–15% of all patients with acute coronary syndrome. ^2^

Typical Wellens syndrome usually presents to the emergency department with pain-free symtomps and normal or slightly elevated troponin level. However, it is important to recognize the electrocardiogram patterns as these patients are at high risk for impending large anterior wall myocardial infarction. Thus, Wellens syndrome has often been considered an STEMI equivalent.

On the other hand, the presence of coronary collateral circulation can change an electrocardiogram picture that should be STEMI due to total or near total occlusion of the left anterior descending artery, turning into an electrocardiogram of Wellens syndrome.

In this case, we present Wellens syndrome in acute total occlusion saved by collateral. Significant collateral circulation is believed to improve clinical outcomes, especially in patients with acute coronary syndrome.

## Case report

A 57-year-old man presented at Dr. Soetomo General Hospital, Surabaya, Indonesia, with sudden chest pain that started half an hour after eating and lasted two hours. He had a history of poorly controlled type 2 diabetes mellitus, poorly controlled hypertension, hypercholesterolemia, and a history of angina three years ago without undergoing a revascularization procedure. There was no social history of smoking nor drinking. He was brought to the emergency department with acute onset of substernal chest pain and diaphoresis. The chest pain started two hours before he was brought to the emergency ward. His chest pain score was four on the visual analogue scale (VAS). Nitroglycerin was administrated then the patient's chest pain disappeared. Physical examination on admission revealed a temperature of 36.7°C, a pulse of 99 beats/min, blood pressure of 170/100 mmHg, respiration of 22/min and oxygen saturation was 95% on room air. He was overweight with a BMI of 29.4 kg/m ^2^. Others physical examination were unremarkable.

### Timeline

**Figure 1.**
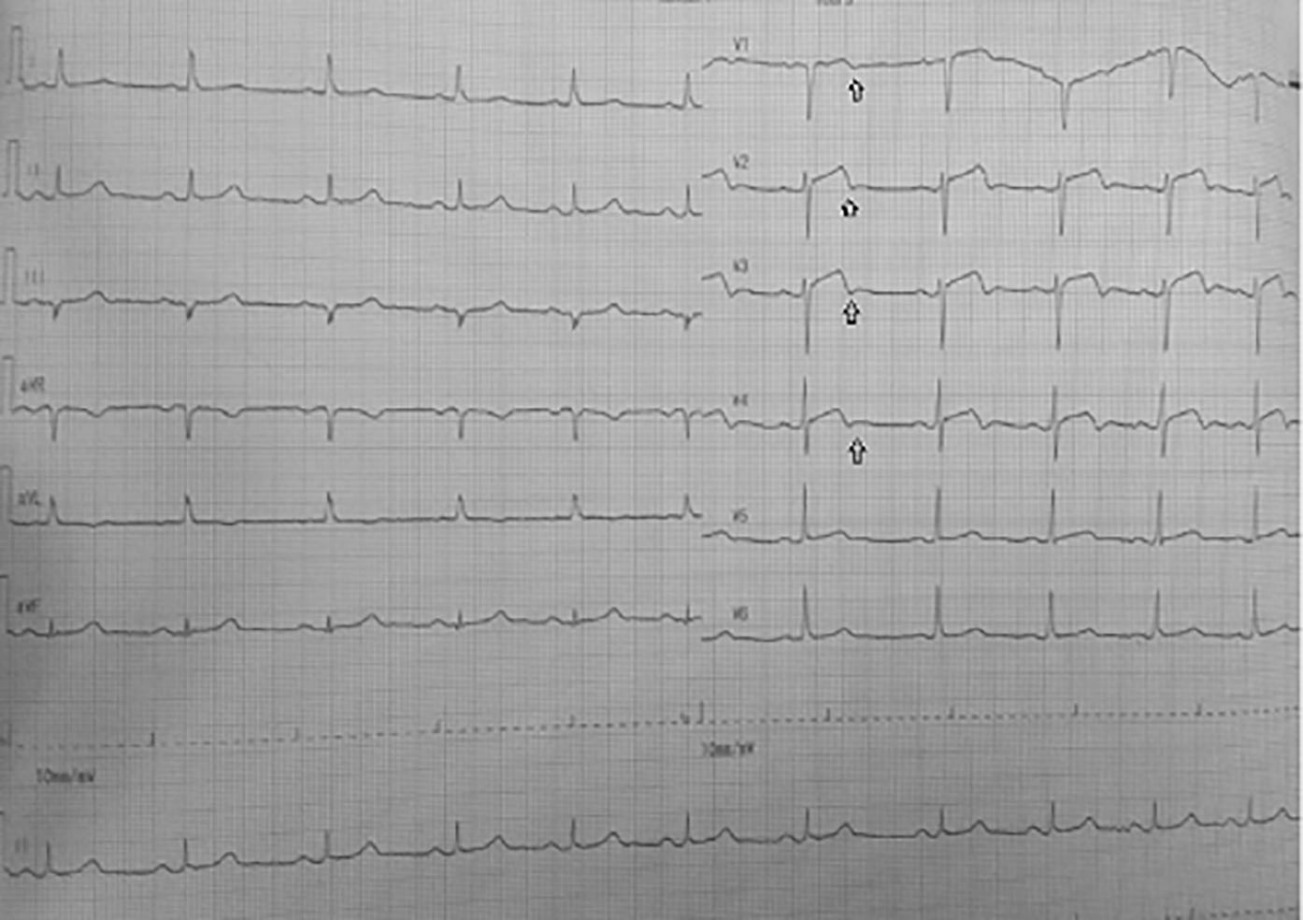
Electrocardiogram: Biphasic T wave in V1–V4 (Wellens syndrome type A) and slight ST elevation in lead V2–V4.

**Figure 2.**
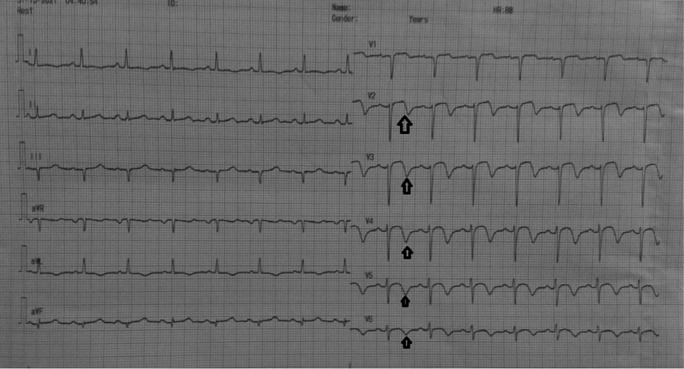
Electrocardiogram: T-wave inversion in leads V2–V5 and slight ST-segment elevation in lead V2–V3.

On day one, the patient was admitted to our emergency ward due to abrupt and sudden onset chest pain with visual analogue scale 4/10, that was not relieved by nitroglycerine. Electrocardiogram showed a biphasic T wave in V2–V4 (type A Wellens) ( Figure 1). The patient was diagnosed with non-ST elevation myocardial infarction. On day two, transthoracic echocardiography revealed regional wall motion abnormality with reduced ejection fraction. Electrocardiogram changed into an anterior acute ST-elevation myocardial infarction. Then, we planned to perform early invasive strategy. Coronary angiography revealed a total occlusion in the middle left anterior descending coronary artery. We performed percutaneous coronary intervention and stenting in the lesion. On day four, the patient improved significantly during the critical period and was transferred to low care. On day five, Electrocardiogram revealed an inverted T wave in V2–V6 (type B Wellens) ( Figure 2), and then the patient was discharged without any sequelae.

### Investigations

Significant laboratory findings suggested significant raised of highly sensitive troponin I (hs-TnI) levels 2,058 ng/ml (normal value: <0.02), HbA1c 8.5% (normal value: <6.5%), and complete blood count, renal function test, liver function test and serum electrolyte were within normal limits. Electrocardiography in the emergency department ( Figure 1) showed an ST segment of less than 1 mm, and there was a biphasic T wave in V2–V4 (type A Wellens) ( Figure 2); echocardiography was therefore performed showed ejection fraction was 48%, there was left ventricular dilatation (left ventricular internal diameter end diastole 6.1cm) and eccentric left ventricular hypertrophy (left ventricular mass index: 132.62 g/m ^2^; relative wall thickness: 0.320); from segmental analysis, it was found that there was hypokinetic in the anteroseptal (basal-mid) region, septal (apical) region; others regions were within normal limits.

**Figure 3.**
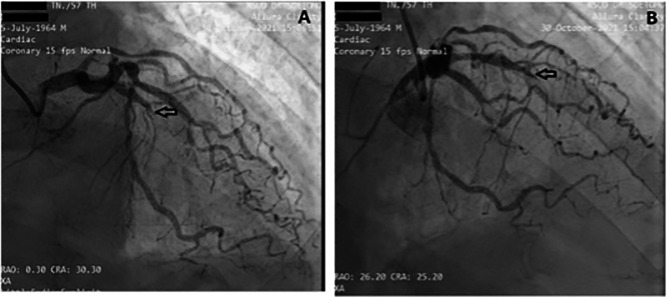
Angiography: A and B LAD projection view showed total occlusion of 100% in the middle LAD. The left circumflex coronary artery (LCx) had non-significant stenosis of 40% at proximal and 65% at distal.

**Figure 4.**
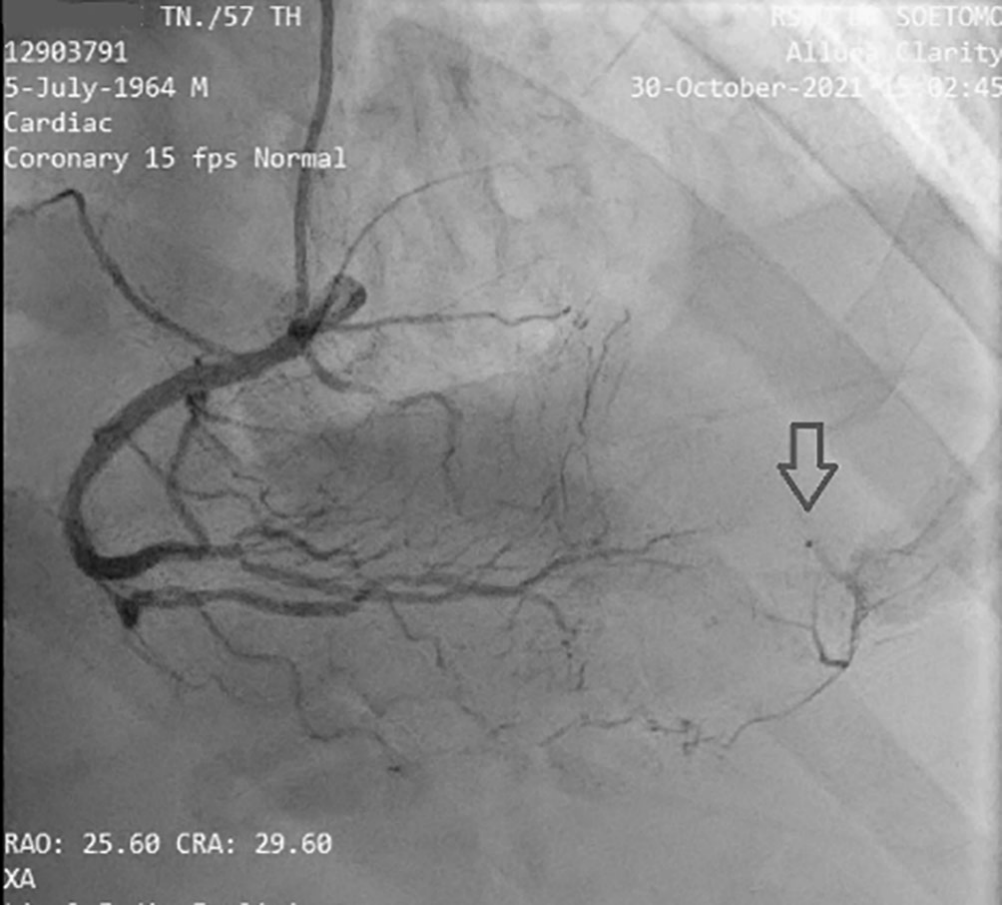
Angiography RCA projection View RAO, CRA. It is revealed that there is grade II collateral from distal right coronary artery to distal left anterior descending coronary artery. (Notes: RAO CRA = right anterior oblique + cranial view, a radiographic projection).

Before the invasive strategy was done, the patient had another electrocardiography eight hours after the first electrocardiogram, based on the second ( Figure 2). The electrocardiogram showed ST-segment elevation changes that were greater than 1 mm. The early invasive strategy ( Figure 3 and Figure 4; Extended data: Video 1 and Video 2) ^3^^,^^4^ showed that the left anterior descending artery had a critical occlusion of 99% in the middle of left anterior descendants coronary artery ( Figure 3). The left circumflex coronary artery had non-significant stenosis of 40% proximal and 65% distal, and right coronary artery had non-significant stenosis of 55% distal. The right coronary artery had Rentrop grade 2 collateral arteries that supply blood to the middle of left anterior descending coronary artery ( Figure 4).

### Differential diagnosis

• Other STEMI equivalent patterns: Hyperacute T-waves, de Winter T-waves, Isolated posterior MI, T waves upright in V1. It is important to remember that an electrocardiogram is not 100% sensitive for coronary occlusion and that the absence of STE does not rule out an MI.
• Persistent juvenile T-wave pattern: patients are typically under 30 years old with asymmetrically inverted T-waves in V1-V3.
• Digitalis effect: diagnosis of digitalis toxicity can be ruled out since there are no history of digitalis consumption.
• Acute myocarditis: no history of viral illness nor influenza-like illness.
• Preexcitation syndromes: no delta waves nor prominent R waves in V1-3, no shortening PR intervals. Concealed pathway can’t be rule out, however, our patient never experiences any tachyarrhythmias episode.
• Later stages of pericarditis: physical examination didn’t find any friction rub. Echocardiography didn’t find any pericardial effusion nor constrictive pattern.
• Right Bundle Branch Block (RBBB): the electrocardiogram shows an inverted T wave, RsR’ in lead V1–V3, QRS duration is >130 ms.
• Pulmonary embolism: has symptoms of chest pain, but the electrocardiogram shows S1Q3T3. It means the presence of an S wave in lead I (indicating a rightward shift of the QRS axis) with a Q wave and T inversion in lead III.
• Cocaine users: has electrocardiogram patterns like Wellens, called “pseudo-Wellens syndrome”, due to vasospasm of the coronary arteries.
• Central nervous system disorders: disorders of the central nervous system usually cause abnormalities in ventricular repolarisation. However in this case, no history of central nervous system trauma, stroke, tumor or infection.

### Treatment

In accordance with the 2020 European Society of Cardiology Guidelines for Acute Coronary Syndrome, ^5^ our patient received an acetylsalicylic acid loading dose of 300 mg followed by 100 mg once daily, high dose statin with Atorvastatin 40 mg once daily at night, Bisoprolol 2.5 mg once daily, Ramipril 5 mg once daily in the morning, Fondaparinux injection 2.5 mg subcutaneous once daily, IV nitroglycerine 20 μ g/minute for hypertension; the patient’s angina subsided. He was also receiving basal insulin bolus for his diabetes. An early invasive strategy was carried out and the patient was then moved to the ICCU.

### Outcome and follow up

Our patient improved significantly during the critical period. After two days in the ICCU, he had no complaints. Based on physical examination, he was afebrile. His heart rate was 70 beats per minute, with blood pressure 123/70 mmHg, respiratory rate 18 breaths per minute, and peripheral oxygen saturation was 95% with a nasal cannula of three litres per minute. Our patient was no longer using nitroglycerine pump and anticoagulant, and he was transferred to the low-care unit. After three days in the hospital, the results of the ECG examination showed an inverted T wave in V2–V6, and then the patient was discharged without any sequelae. After one month, the patient was well controlled for his disease without any sequelae.

## Discussion

Current STEMI criteria leaves the clinician restricted in early-decision making processes if specific measurements are not met. Under the STEMI/NSTEMI paradigm, up to 30% of patients we classify as NSTEMI are consistently found to have missed acute coronary occlusion. ^6^ Although these patients often end up receiving intervention at a later stage (24-72 hours) into admission, it is usually too late to salvage ischaemic or infarcted tissue and they are exposed to significant increases in morbidity and mortality. The term “STEMI-equivalent” is outdated. Patients with acute occlusion not meeting STEMI criteria may be an underserved, underidentified subgroup of acute coronary syndrome patients who would benefit from emergent intervention. ^7^

Therefore, in 2018, Meyers, Weingart and Smith introduced the concept of Occlusion Myocardial Infarction through the Manifesto. ^8^ Since this, there has been gaining awareness that ST-elevation on electrocardiogram is most likely an unreliable tool for detecting patients that will benefit from PCI, and that a shift is required to a more reliable paradigm for detecting acute coronary occlusion. Occlusion Myocardial Infarction is a branch of the acute coronary syndrome algorithm representing near or total occlusion with insufficient collateral circulation causing active infarction. On the other hands, Non-Occlusion Myocardial Infarction (NOMI) explains no occlusion nor sufficient collateral circulation to avoid active infarction. Patients with Occlusion Myocardial Infarction are those with benefit from emergent reperfusion therapy, and in which the benefits outweigh the risks of this invasive procedure. ^9^

Wellens electrocardiogram pattern is commonly found in a patient with total or near total occlusion in the proximal left anterior descending coronary artery. It is commonly associated with NSTE-ACS, followed by new onset angina CCS III-IV or crescendo angina, without increased cardiac markers. ^10^ The spontaneous transformation from Wellens type A ECG pattern into Wellens type B ECG pattern is a rare case. It reflects a pattern of electrocardiogram that sometimes changes due to occlusion and spontaneous reperfusion from the collateral coronary artery. Type A and type B Wellens particular for critical, proximal stenosis of the left anterior descending (LAD) coronary artery. ^11^ Criteria for diagnosing Wellens syndrome include all of the following ^12^^,^^13^:

• Type A Wellens describes a pattern of electrocardiogram that shows biphasic T wave in the lead V2–V4
• Type B Wellens describes a pattern of electrocardiogram that shows T inverted in the lead V2–V4
• History of angina
• An electrocardiogram without Q wave
• Normal or minimally elevated troponin levels
• ST segment isoelectric or minimally elevated (<1 mm)

In this case, the first electrocardiogram showed a biphasic T wave in lead V1–V4 (Wellens type A) and it was minimally elevated (<1 mm). After a few hours before the invasive procedure, the second ECG showed a change in ST-segment elevation of more than 1 mm. In this case, the patient's ECG and troponin level showed Wellens syndrome. However, after a few hours the patient's electrocardiogram showed dynamic changes, namely an increase in elevation in the ST segment (>1 mm). An abnormal T wave ischemic pattern may persist, remaining between hours to weeks, even when the patient is asymptomatic without chest pain. ^7^ T wave abnormalities can be normalized or evolved into ST-segment elevation in the symptomatic patient with Wellens syndrome. The mechanism responsible for these electrocardiogram findings is repolarization heterogeneity resulting from reperfusion of a briefly occluded left anterior descending. This could explain the evolution of T-wave modifications. ^14^^,^^15^ In this case, there was complete occlusion in the middle left anterior descendents coronary artery, but the patient received collateral artery supply from the right coronary artery so that the electrocardiogram feature mimicked the critical occlusion picture in the proximal left anterior descending coronary artery.

The collateral of the coronary artery is classified into five grades. Collateral grade 0 reveals no flow between the collateral of the coronary artery. This condition can occur when several collateral arteries are visible yet not angiographically apparent at any other time. Collateral grade 1 flow reveals a barely apparent collateral coronary artery. Sometimes, there might be an unclear connection with the significant epicardial coronary artery. Collateral grade 2 flow reveals a moderately opaque collateral coronary artery but it was only present through 75% of the cardiac cycle. Collateral grade 3 flow reflects a well-opacified collateral coronary artery while the column of dye is well defined (i.e., >0.5 mm in diameter), but it was <0.7 mm wide throughout most of its length. Collateral grade 4 flow mimics collateral grade 3. The collateral is very well opacified, fills antegrade, and is very large. It was >0.7 mm in diameter throughout its length. ^16^^,^^17^

The cardiac catheterization results found that a collateral artery originating from the right coronary artery supplied the mid-distal left anterior descending coronary artery. Therefore the heart muscle that should have been severely damaged due to not getting blood supply from the left anterior descending could still survive due to the presence of the collateral artery. Previous data has established that enough collaterals can prevent ischemia and directly induce spontaneous reperfusion in one-third of NSTE-ACS patients. Spontaneous reperfusion with well-developed coronary collateral circulation is associated with a better prognosis in cardiovascular mortality reduction. ^18^

In ischemic conditions, collateral circulations are a good predictor of prognosis. Collateral circulation may support cardiac function and decrease cardiac mortality and morbidity rate. Collateral circulations are inter arterial anastomoses that exist neonatally and grow more significant because of many mechanisms like cytokines and growth factors induced by shear stress and ischemia and pressure gradient changes. ^19^ The collateral circulation artery may appear several weeks after occlusion. ^18^

## Conclusions

This case shows Wellens syndrome, which can change into ST-elevation myocardial infarction. Wellens can also occur in the left anterior descending artery that is in total occlusion but gets its blood supply from a branch of the right coronary collateral artery. Based on this case report, an early invasive strategy is recommended, with favourable clinical outcomes. Failing to recognize Wellens syndrome or T-wave dynamic changes on electrocardiogram could lead to significant mortality and morbidity, such as underestimating the seriousness of the electrocardiogram finding in a pain-free patient, failing to properly admit or consult cardiology leads to delay percutaneous coronary intervention, ordering unnecessary stress test which may provoke a large anterior wall myocardial infarction.

### Consent

Written informed consent for publication of their clinical details and clinical images was obtained from the patient.

## Data availability

### Underlying data

All data underlying the results are available as part of the article and no additional source data are required.

### Extended data

Figshare: Extended data for ‘Case Report: Wellens syndrome in acute total occlusion saved by collateral’.

This project contains the following extended data:

Video 1: Angiography revealed total occlusion at mid left anterior descending coronary artery Video 1: Angiography revealed total occlusion at mid left anterior descending coronary artery Copyright: © 2024 Alsagaff MY et al. 2024

Video 2: Angiography revealed collateral from distal right coronary artery to distal left anterior descending coronary artery Video 2: Angiography revealed collateral from distal right coronary artery to distal left anterior descending coronary artery Copyright: © 2024 Alsagaff MY et al. 2024

Data are available under the terms of the Creative Commons Attribution 4.0 International license (CC-BY 4.0).
