# Supplementary material for: Case Report: Wellens syndrome in acute critical coronary occlusion saved by collateral
Source: F1000Res. 2024 Jul 5;11:1486. Originally published 2022 Dec 12. [Version 3] doi: 10.12688/f1000research.125820.3 (PMC11301133; doi:10.12688/f1000research.125820.3)
Supplement: Peer review history

# Reviewer report: Reviewer response for version 3

**DOI:** 10.5256/f1000research.168673.r299641

**Published:** 2024-08-05

**Authors:** Dhan Bahadur Shrestha

**Affiliations:** 1 Department of Internal Medicine, Mount Sinai Hospital, Chicago, Illinois, USA

The manuscript is much better and should be indexed with proofreading and production editing.

Are enough details provided of any physical examination and diagnostic tests, treatment given and outcomes?

Partly

Is the case presented with sufficient detail to be useful for other practitioners?

Partly

Is sufficient discussion included of the importance of the findings and their relevance to future understanding of disease processes, diagnosis or treatment?

Partly

Is the background of the case’s history and progression described in sufficient detail?

Partly

Reviewer Expertise:

General Cardiology, Arrythmia and electrophysiology, Sudden cardiac death/Cardiac arrest

I confirm that I have read this submission and believe that I have an appropriate level of expertise to confirm that it is of an acceptable scientific standard.

# Reviewer report: Reviewer response for version 2

**DOI:** 10.5256/f1000research.167065.r285149

**Published:** 2024-06-27

**Authors:** Zahid Khan

**Affiliations:** 1 Barts Health NHS Trust, London, UK

Thank you for inviting me to review the revised version. Please make the following changes to the article.

 He was overweight with a pained grunt and he occasionally clutched his chest. Others physical examination were unremarkable. **The overweight part should come in the past medical history.**

 Differential diagnosis should make sense and should not be just random differentials. Please rewrite these and the differentials ideally should be in a paragraph form.

Are enough details provided of any physical examination and diagnostic tests, treatment given and outcomes?

Partly

Is the case presented with sufficient detail to be useful for other practitioners?

Partly

Is sufficient discussion included of the importance of the findings and their relevance to future understanding of disease processes, diagnosis or treatment?

Partly

Is the background of the case’s history and progression described in sufficient detail?

Partly

Reviewer Expertise:

Intervention cardiology and General internal Medicine

I confirm that I have read this submission and believe that I have an appropriate level of expertise to confirm that it is of an acceptable scientific standard.

# Response

**Published:** 2024-06-30

**Authors:** Ricardo Adrian Nugraha

**Affiliations:** Airlangga University, Indonesia

Thank you very much for taking the time to share your suggestions with us.

# Reviewer report: Reviewer response for version 2

**DOI:** 10.5256/f1000research.167065.r285150

**Published:** 2024-06-18

**Authors:** Dhan Bahadur Shrestha

**Affiliations:** 1 Department of Internal Medicine, Mount Sinai Hospital, Chicago, Illinois, USA

This version is better; however, there are still many instances requiring improvements. Prior comments regarding writing have not been totally addressed, and the writing needs further revisions. 

 For example,  "It's important important to recognize", important is to repetition by mistake in the abstract!

 "He was overweight with a pained grunt and he occasionally clutched his chest. " not fully comprehendible 

 Occlusion Myocardial Infarction (OMI) abbreviated with full form in multiple instances, its recommended to use full form with abbreviations in first use in body of manuscript and only abbreviations in subsequent use. Other similar cases include ECG, LAD, RCA, etc

 Thank you

Are enough details provided of any physical examination and diagnostic tests, treatment given and outcomes?

Partly

Is the case presented with sufficient detail to be useful for other practitioners?

Partly

Is sufficient discussion included of the importance of the findings and their relevance to future understanding of disease processes, diagnosis or treatment?

Partly

Is the background of the case’s history and progression described in sufficient detail?

Partly

Reviewer Expertise:

General Cardiology, Arrythmia and electrophysiology, Sudden cardiac death/Cardiac arrest

I confirm that I have read this submission and believe that I have an appropriate level of expertise to confirm that it is of an acceptable scientific standard, however I have significant reservations, as outlined above.

# Response

**Published:** 2024-06-30

**Authors:** Ricardo Adrian Nugraha

**Affiliations:** Airlangga University, Indonesia

**Reviewer Comment:**

 This version is better; however, there are still many instances requiring improvements. Prior comments regarding writing have not been totally addressed, and the writing needs further revisions. 

**Author response:** Thank you for your help. We’ve got the manuscript better and better from your feedback and suggestions. We really appreciate your perspective and will take it into consideration.

**Reviewer Comment:**

 For example,  "It's important important to recognize", important is to repetition by mistake in the abstract!

**Author response:** Thank you for your feedback, we deleted that repeated word.

**Reviewer Comment:**

 "He was overweight with a pained grunt and he occasionally clutched his chest. " not fully comprehendible 

**Author response:** Thank you for your feedback, we replaced it with “He was overweight with a BMI of 29.4 kg/m ^2^“

**Reviewer Comment:**

 Occlusion Myocardial Infarction (OMI) abbreviated with full form in multiple instances, its recommended to use full form with abbreviations in first use in body of manuscript and only abbreviations in subsequent use. Other similar cases include ECG, LAD, RCA, etc.

**Author response:** Thank you for your suggestion. Since OMI is popular for the abbreviation of Old Myocard Infarct, then we decided not to abbreviate the term of Occlusion Myocardial Infarction.

 Once again, thank you for taking the time to share your suggestions with us.

# Reviewer report: Reviewer response for version 1

**DOI:** 10.5256/f1000research.138167.r263004

**Published:** 2024-05-09

**Authors:** Dhan Bahadur Shrestha

**Affiliations:** 1 Department of Internal Medicine, Mount Sinai Hospital, Chicago, Illinois, USA

Thank you for offering this opportunity to review the manuscript by Alsagaff et al. titled "Case Report: Wellens syndrome in acute total occlusion saved by collateral." It is an interesting topic, and I carefully reviewed the manuscript.

 General Comments

 1. As a rule of writing, it is recommended not to abbreviate if the abbreviation is not used more than once. The abstract and manuscript sections are separate sections; the abbreviations/acronyms need the same consideration. The use of LAD/ICCU in the abstract is unnecessary. However, in the manuscript body, ECG is not abbreviated on its first use. Authors need to be careful in considering the same principle throughout the manuscript

 2. Some written statements are contradictory. The abstract mentions "...near total occlusion in the middle of the left anterior descending artery (LAD) with collateral from the right coronary artery. " However, the case report section mentions total occlusion. For uniformity, authors need to consider the same principle throughout the manuscript. 

 3. I agree with general comments in terms of writing, as suggested by another reviewer. The authors need more background discussion about Wellen syndrome and its equivalent STEMI and perhaps consideration of a new MI classification considering vascular occlusion (OMI vs. N-OMI). It seems the authors are more so focused on collateral circulation in the discussion, which may be less relevant in mentioning detailed classifications of collaterals in half of the discussion section.

 4. It's unclear to me whether the authors reported HS-troponin or just troponin. The AHA 2021 chest pain guideline recommends an HS-troponin assay. 

 5. The relevance of this paragraph "The patient felt that his treatment at the hospital was excellent and fast, especially when he felt chest pain. After the medication was taken, his chest pain gradually disappeared. The patient was also very grateful for the direct action of inserting a catheter into the heart in less than 24 hours. The patient hopes that the publication of cases of diseases like his will help paramedics handle them in the future." is uncertain to me, rather more objective mention of patient care timeline (door to cath lab/balloon) and its alignment with recent 2018 ESC or 2021 AHA/ACC revascularization guideline would be more appropriate.

Are enough details provided of any physical examination and diagnostic tests, treatment given and outcomes?

Partly

Is the case presented with sufficient detail to be useful for other practitioners?

Partly

Is sufficient discussion included of the importance of the findings and their relevance to future understanding of disease processes, diagnosis or treatment?

Partly

Is the background of the case’s history and progression described in sufficient detail?

Partly

Reviewer Expertise:

General Cardiology, Arrythmia and electrophysiology, Sudden cardiac death/Cardiac arrest

I confirm that I have read this submission and believe that I have an appropriate level of expertise to state that I do not consider it to be of an acceptable scientific standard, for reasons outlined above.

# Response

**Published:** 2024-05-25

**Authors:** Ricardo Adrian Nugraha

**Affiliations:** Airlangga University, Indonesia

1. As a rule of writing, it is recommended not to abbreviate if the abbreviation is not used more than once. The abstract and manuscript sections are separate sections; the abbreviations/acronyms need the same consideration. The use of LAD/ICCU in the abstract is unnecessary. However, in the manuscript body, ECG is not abbreviated on its first use. Authors need to be careful in considering the same principle throughout the manuscript

**Authors response:** thank you for your very constructive feedback. We have replaced any abbreviations with the full words.

 2. Some written statements are contradictory. The abstract mentions "...near total occlusion in the middle of the left anterior descending artery (LAD) with collateral from the right coronary artery. " However, the case report section mentions total occlusion. For uniformity, authors need to consider the same principle throughout the manuscript. 

**Authors response:** thank you for your critical observation. We have reviewed again the angiography video and found it was critical occlusion (99% stenosis).

 3. I agree with general comments in terms of writing, as suggested by another reviewer. The authors need more background discussion about Wellen syndrome and its equivalent STEMI and perhaps consideration of a new MI classification considering vascular occlusion (OMI vs. N-OMI). It seems the authors are more so focused on collateral circulation in the discussion, which may be less relevant in mentioning detailed classifications of collaterals in half of the discussion section.

**Authors response:** thank you for your feedback. We have added several background of Wellens syndrome in the Introduction section. We have added the reason to a new MI classification considering vascular occlusion (OMI vs. N-OMI) in the Discussion section.

 4. It's unclear to me whether the authors reported HS-troponin or just troponin. The AHA 2021 chest pain guideline recommends an HS-troponin assay. 

**Authors response:** thank you for your questions. It was HS-troponin I.

 5. The relevance of this paragraph "The patient felt that his treatment at the hospital was excellent and fast, especially when he felt chest pain. After the medication was taken, his chest pain gradually disappeared. The patient was also very grateful for the direct action of inserting a catheter into the heart in less than 24 hours. The patient hopes that the publication of cases of diseases like his will help paramedics handle them in the future." is uncertain to me, rather more objective mention of patient care timeline (door to cath lab/balloon) and its alignment with recent 2018 ESC or 2021 AHA/ACC revascularization guideline would be more appropriate.

**Authors response:** after reviewing it carefully, we decided to delete this irrelevant paragraph.

# Reviewer report: Reviewer response for version 1

**DOI:** 10.5256/f1000research.138167.r263011

**Published:** 2024-04-13

**Authors:** Zahid Khan

**Affiliations:** 1 Barts Health NHS Trust, London, UK

The introduction should provide some more information about Wellens syndrome and why early intervention is important.

 Please exclude any patient identifiable information such as location of the patient/hospital provided in the case report section.

 Please use the term poorly controlled rather uncontrolled for both hypertension and diabetes.

 I would suggest to use either recent history or history of angina three years ago rather than using both.

 There was no history of cocaine use (This is not required and it should be part of the social history along with smoking and drinking)

 On physical examination on the emergency ward (in the emergency ward , oxygen saturation of 95% on room air)

 The trop rise of this level does not really fit with Wellens syndrome. Wellens syndrome patients tend to have mild trop rise

 The differential diagnosis are poorly constructed and please include more relevant differential diagnosis.

 General comments.

 The case report is about a patient presenting with chest pain and the authors claim that the patient had wellens syndrome. The case report has significant grammatical errors that need rectifying. The presentation lacks flow and is disjointed. The high level of trop does not really fit with wellens syndrome type picture. Please remove all patient's identifiable information from the case report. The introduction and case presentation sections need significant revision.

Are enough details provided of any physical examination and diagnostic tests, treatment given and outcomes?

Partly

Is the case presented with sufficient detail to be useful for other practitioners?

Partly

Is sufficient discussion included of the importance of the findings and their relevance to future understanding of disease processes, diagnosis or treatment?

Partly

Is the background of the case’s history and progression described in sufficient detail?

Partly

Reviewer Expertise:

Intervention cardiology and General internal Medicine

I confirm that I have read this submission and believe that I have an appropriate level of expertise to state that I do not consider it to be of an acceptable scientific standard, for reasons outlined above.

# Response

**Published:** 2024-05-25

**Authors:** Ricardo Adrian Nugraha

**Affiliations:** Airlangga University, Indonesia

The introduction should provide some more information about Wellens syndrome and why early intervention is important.

**Authors response:** thank you for your suggestion. We have provided some more information about Wellens syndrome in the Introduction section. We have provided the reason why early intervention is important in the Conclusion section.

 Please exclude any patient identifiable information such as location of the patient/hospital provided in the case report section.

**Authors response:** thank you for your constructive feedback. We have removed any patient identifiable information In our 2 videos.

 Please use the term poorly controlled rather uncontrolled for both hypertension and diabetes.

**Authors response:** thank you for your suggestion. We have used the term poorly controlled hypertension and diabetes.

 I would suggest to use either recent history or history of angina three years ago rather than using both.

**Authors response:** thank you for your suggestion. We have used the term history of angina three years ago.

 There was no history of cocaine use (This is not required and it should be part of the social history along with smoking and drinking)

**Authors response:** thank you for your suggestion. We have used the term social history of smoking or drinking.

 On physical examination on the emergency ward (in the emergency ward , oxygen saturation of 95% on room air)

**Authors response:** thank you for your suggestion. We have used the term as you have suggested.

 The trop rise of this level does not really fit with Wellens syndrome. Wellens syndrome patients tend to have mild trop rise

**Authors response:** thank you for your suggestion. Unfortunately, our case has significant raised of troponin level which may be unsuitable for Wellens syndrome. We have tried to search for any literatures regarding cardiac enzyme among Wellens syndrome, and found that troponin may be falsely reassuring in patients with Wellens syndrome. Only 12% of patients with Wellens syndrome had elevated cardiac enzymes, and these elevations were less than twice the upper limit of normal. Increase of cardiac enzymes in our case may indicates that cardiac myonecrosis have already been occurred at the time of diagnosis.

 The differential diagnosis are poorly constructed and please include more relevant differential diagnosis.

**Authors response:** thank you for your suggestion. We have added several differential diagnosis: Hyperacute T-waves, de Winter T-waves, Isolated posterior MI, T waves upright in V1, Persistent juvenile T-wave pattern, Digitalis toxicity, Acute myocarditis, Preexcitation syndromes, Later stages of pericarditis, and Central nervous system disorders.

 General comments.

 The case report is about a patient presenting with chest pain and the authors claim that the patient had wellens syndrome. The case report has significant grammatical errors that need rectifying.

**Authors response:** thank you for your suggestion. We tried to correct several grammatical errors in our literatures.

 The presentation lacks flow and is disjointed. The high level of trop does not really fit with wellens syndrome type picture.

**Authors response: **thank you for your suggestion. Unfortunately, our case has significant raised of troponin level which may be unsuitable for Wellens syndrome. We have tried to search for any literatures regarding cardiac enzyme among Wellens syndrome, and found that troponin may be falsely reassuring in patients with Wellens syndrome. Only 12% of patients with Wellens syndrome had elevated cardiac enzymes, and these elevations were less than twice the upper limit of normal. Increase of cardiac enzymes in our case may indicates that cardiac myonecrosis have already been occurred at the time of diagnosis.

 Please remove all patient's identifiable information from the case report.

**Authors response:** thank you for your constructive feedback. We have removed any patient identifiable information In our 2 videos.

 The introduction and case presentation sections need significant revision.

**Authors response:** thank you for your suggestion. We have provided some more information about Wellens syndrome in the Introduction section.
